# Effect of Doxycycline Use in the Early Broiler Production Cycle on the Microbiome

**DOI:** 10.3389/fmicb.2022.885862

**Published:** 2022-07-07

**Authors:** Genevieve Greene, Leonard Koolman, Paul Whyte, Catherine Burgess, Helen Lynch, Aidan Coffey, Brigid Lucey, Lisa O’Connor, Declan Bolton

**Affiliations:** ^1^Teagasc Food Research Centre, Dublin, Ireland; ^2^School of Veterinary Medicine, University College Dublin, Dublin, Ireland; ^3^Department of Agriculture, Food and the Marine, Backweston, Ireland; ^4^Department of Biological Sciences, Munster Technological University, Cork, Ireland; ^5^Food Safety Authority of Ireland, Dublin, Ireland

**Keywords:** broilers, 16S rRNA amplicon sequencing, gastrointestinal tract microbiota, antibiotic treatment, Firmicutes/Bacteroidetes ratio

## Abstract

16S rRNA amplicon sequencing was used to investigate changes in the broiler gastrointestinal tract (GIT) microbiota throughout the rearing period and in combination with antibiotic treatment. Thirty birds (from a commercial flock) were removed at multiple points throughout the rearing period on days 13, 27, and 33, euthanised, and their GIT aseptically removed and divided into upper (the crop, proventriculus, and the gizzard), middle (the duodenum, jejunum, and ileum) and lower (the large intestine, the caeca, and the cloaca) sections. In a separate commercial flock, on the same farm with similar husbandry practices and feed, doxycycline (100 mg/ml per kg body weight) was administered in drinking water between day 8 and 12 (inclusive) of the production cycle. Birds were removed on days, 13, 27, and 33 and GIT samples prepared as above. The contents of three merged samples from each GIT section were pooled (*n* = 60), the DNA extracted and analysed by 16S rRNA amplicon metagenomic sequencing and analysed. Major changes in the broiler microbiota were observed as the birds aged particularly with the Firmicutes/Bacteroidetes ratio (F:B) of the lower GIT. Moreover, Chao1, ACE, and Shannon indices showed the antibiotic treatment significantly altered the microbiota, and this change persisted throughout the rearing period. Further research is required to investigate the effect of these changes on bird performance, susceptibility to infections and *Campylobacter* carriage.

## Introduction

Poultry is the most consumed meat in the world, with ~14.7 kg/capita consumed annually (OECD, 2021). To satisfy this demand, aviculture systems have become very effective at converting feed into muscle protein (Clavijo and Flórez, 2018). The microbiota of the gastrointestinal tract (GIT) has an important function in this system as they release nutrients from feed, provide immunity and promote gut health. Thus, an improved understanding of the broiler microbiome could provide the scientific basis for enhanced broiler health, wellbeing and productivity (Clavijo and Flórez, 2018; Glendinning et al., 2019). However, there is currently a dearth of information on the bacterial composition of the broiler gut and how this varies depending on the location within the GIT.

Firmicutes, Cyanobacteria, Actinobacteria, Proteobacteria, and Bacteroidetes are abundant in the broiler GIT (Dragana et al., 2013; Feye et al., 2020), but the relative abundance of the different genera vary considerably depending on the section of the GIT (Dragana et al., 2014; Rychlik, 2020). Lactobacilli, for example, are known to be major colonisers of the upper GIT of broilers, where they digest starch and ferment lactate (Clavijo and Flórez, 2018).

Several factors affect the microbial composition at different regions of the broiler GIT, including breed type, age, gender, diet/feed type, antibiotic use, broiler house hygiene, maternal factors, and litter type (Clavijo and Flórez, 2018; Kers et al., 2018). Antibiotics effectively prevent and treat avian diseases in broilers and may also promote growth, although they have been banned for the latter use in the European Union since 2006 and in the United States of America since 2017 (Roth et al., 2019). Antibiotic treatments suppress pathogens (Maki et al., 2019) but may also cause a dysbiosis that adversely effects systemic energy metabolism (Le Roy et al., 2019). Villous atrophy, diminished thickness of the tunica muscularis, and an increase in T lymphocyte infiltration of the gut mucosa have also been associated with microbiota disturbance in the broiler GIT after antibiotic treatment (Pereira et al., 2019). Moreover, antibiotics reduce both the stability and composition of the microbiota. *Lactobacillus* spp., for example (essential for immunity, metabolism, and bird growth), may decrease in treated birds depending on the antimicrobial used (Gao et al., 2017; Clavijo and Flórez, 2018; Haberecht et al., 2020).

Doxycycline was the antibiotic used on the broiler farm in this study. It is a broad-spectrum tetracycline antibiotic that is widely used in farming (Pavlova, 2015; Blau et al., 2019). However, specific information on how it affects the broiler microbiota is lacking.

Broilers are typically provided with three feed types throughout their life cycles (starter, grower, and finisher feeds) which may affect the GIT microbiota (Saleh et al., 1997; Greene et al., 2020). Starter feeds are high in protein and low in carbohydrates, with protein to carbohydrates ratios reversed in finisher feeds (Greene et al., 2020). Compositional variations in the successive formulations can affect different regions of the broiler GIT (Haberecht et al., 2020). Simple digestible carbohydrates have a strong effect on the small intestine as they are quickly absorbed and utilised by the microbiota. Dietary fibres, like non-digestible carbohydrates, non-starch polysaccharides, non-digestible oligosaccharides, and resistant starch, travel to the lower intestines and caeca, largely undigested, promoting the growth of beneficial bacteria such as *Bifidobacterium, Lactobacillus* spp., *Akkermansia muciniphila,* and *Faecalibacterium prausnitzii*. Moreover, the metabolic products released by these bacteria during the digestion of dietary fibre are beneficial to the host’s intestinal and general health (Haberecht et al., 2020). While previous studies have examined the broiler gut microbiota, data on bacterial composition at different stages of the production cycle as the feed type changes and the birds get older is lacking.

The objectives of this study were to provide information on the different bacterial phyla and genera in the upper, middle, and lower sections of the broiler GIT at different stages in the production cycle and to investigate the effect of early antibiotic treatment in the production cycle on the broiler GIT microbiota.

## Materials and Methods

### Study Design

The two flocks used in this study were raised in separate broiler houses on the same commercial farm in County Monaghan in Ireland. The treated flock received Doxivex, a commercial form of the antibiotic doxycycline, used to treat broilers for pasteurellosis, caused by *Pasteurella multocida* and respiratory infections caused by *Ornithobacterium rhinotracheale*. It was administered in drinking water at a concentration of 100 mg/ml per kg body weight, for 5 days between days 8 and 12, inclusive. The untreated birds (control) did not receive any antibiotics. Apart from this, all other husbandry practices, including feed were similar. Exactly 30 birds were removed from each flock on days 13, 27, and 33, midway through their starter (provided from day 1–14), grower (day 15–27), and finisher feed (day 28–42) diets. These birds were transported to a veterinary practice, euthanised *via* cervical dislocation and their gastrointestinal tract (GIT) aseptically removed. GIT samples were transported under chilled conditions to our laboratory (a journey of ~90 min), where each was immediately divided into upper (the crop, proventriculus, and the gizzard), middle (the duodenum, jejunum, and ileum), and lower (the large intestine, caeca, and cloaca) segments and their contents removed. Three of the same sample type were pooled (*n* = 180) and immediately frozen at −80°C. Thus, there were 18 different GIT section-treated/untreated-sampling time combinations as follows; upper GIT samples; [1] antibiotic treated birds at day 13 (UT13); [2] treated birds at day 27 (UT27); [3] treated birds at day 33 (UT33); [4] untreated birds at day 13 (UU13); [5] untreated birds at day 27 (UU27), and [6] untreated birds at day 33 (UU33); middle GIT samples; [7] treated birds at day 13 (MT13); [8] treated birds at day 27 (MT27); [9] treated birds at day 33 (MT13); [10] untreated birds at day 13 (MU13); [11] untreated birds at day 27 (MU27), and [12] untreated birds at day 33 (MU33); lower GIT samples; [13] treated birds at day 13 (LT13); [14] treated birds at day 27 (LT27); [15] treated birds at day 33 (LT33); [16] untreated birds at day 13 (LU13); [17] untreated birds at day 27 (LU27), and [18] untreated birds at day 33 (LU33; Table 1).

**Table 1:** The 18 different GIT section-treated/untreated-sampling time combinations in this study.

Time (day)	Untreated			Treated		
	Upper	Middle	Lower	Upper	Middle	Lower
13	UU13	MU13	LU13	UT13	MT13	LT13
27	UU27	MU27	MU27	UT27	MT27	LT27
33	UU33	MU33	LU33	UT33	MT33	LT33

The upper consists of the crop, proventriculus, and the gizzard, the middle consists of the duodenum, the jejunum, and the ileum, and the lower consists of the large intestine, the caeca, and the cloaca.

### DNA Extraction, Quantification, Qualification, Purification, and Sequencing

DNA was extracted from samples using the DNeasy PowerSoil Pro kit (Qiagen, Manchester, United Kingdom). The quality and concentration was measured using the NanoDrop spectrophotometer (NanoDrop 1,000, ThermoFisher Scientific, Dublin). The DNA was then diluted, in sterile water, to a final concentration of 1 ng/μl. Next, amplicon generation was performed at Novogene Bioinformatics Technology Co., Ltd. (Beijing, China), where distinct 16S rRNA genes of interest were amplified using the primer set 16S V3/V4: 515F-806R (amplicon size 466 bp) with the barcode included in the PCR product (Yu et al., 2005). PCR reactions were performed using Phusion® High-Fidelity PCR Master Mix (New England Biolabs, United States). PCR products were mixed with 1X loading buffer containing SYBR green in a 1:1 ratio and electrophoresed on a 2% agarose gel. Samples with a bright band at the 400–450-bp marker were excised using the Qiagen Gel Extraction Kit (Qiagen, Germany). DNA libraries were prepared using the NEBNext^®^ Ultra^™^ DNA Library Prep Kit (New England Biolabs, United States) for Illumina and quantified *via* Qubit and qPCR. Libraries were analysed on the Illumina NovaSeq 6000 platform.

### Statistical Analysis

#### Processing of Sequence Data

Paired end reads were assigned to samples using the unique barcodes incorporated into each amplicon. Following this the barcode and primer sequences were excised and truncated paired end reads were merged using FLASH (V1.2.7; Magoč and Salzberg, 2011). Quality filtering of these raw tags was performed using the Qiime (V1.7.0) quality controlled process, yielding high quality clean tags (Caporaso et al., 2010; Bokulich et al., 2013). The SILVA (release 138) database was used as a reference database for comparison of high quality tags using the UCHIME algorithm to detect chimera sequences, which were removed, producing the effective tags (Edgar et al., 2011; Haas et al., 2011).

#### OTU Cluster and Taxonomic Annotation

Uparse software (v7.0.1090) was used to perform sequence analysis on all effective tags (Edgar, 2013). Sequences with ≥97% similarity were assigned to the same Operational Taxonomic Unit (OTU) and a representative sequence for each OTU was screened, as follows, for further annotation. Each representative sequence was screened *via* Qiime (Version 1.7.0) using the Mothur pipeline and SILVA’s SSUrRNA database, providing species annotation at each taxonomic rank (Altschul et al., 1990; Wang et al., 2007; Quast et al., 2013). The phylogenetic relationship of all OTUs was obtained using MUSCLE (version 3.8.31; Edgar, 2004). This data was then normalised using the sequence number corresponding to the sample with the least sequences as a standard. Alpha and beta diversity analyses were performed on this normalised data.

#### Alpha Diversity, Beta Diversity, LEfSe Analysis, and Statistical Analysis

The complexity and diversity of samples was analysed using three alpha diversity indices: ACE, Chao1, and Shannon. All diversities were calculated using Qiime (version 1.7.0) and visualised with R software (version 2.15.3). Differences in the species complexity of samples was analysed *via* beta diversity utilising both weighted and unweighted unifrac, using Qiime software (version 1.7.0). Principal component analysis (PCA) was used to perform cluster analysis. The dimension of the original variables was reduced using the FactoMineR and ggplot2 packages in R software (version 2.15.3). Principal Coordinate Analysis (PCoA) was then used to obtain principal coordinates and visualise the complex multidimensional data. A distance matrix of weighted and unweighted unifrac data was transformed into a new set of orthogonal axes where the maximum factor was demonstrated by first principal coordinate, and the second maximum factor by the second principal coordinate, *et cetera*. The WGCNA, stat, and ggplot2 packages in R software (version 2.15.3) were used to display PCoA analysis. Linear discriminant analysis Effect Size (LEfSe) software was used to conduct LEfSe analysis. A permutation test was used to calculate *p*-value, (White et al., 2009) and *T*-test and drawing was conducted using R software. The relative abundance of the top five phyla and genera at each time point and in each GIT section were compared between treated and untreated birds using the paired Wilcoxon test and *p*-values were adjusted using the Bonferroni correction.

### Ethical Approval

The experimental design and protocols in this study were approved by the Teagasc Animal Ethics Committee on the 27th May 2021 (TAEC2021-305).

## Results

### OTU Identification and Taxonomic Annotation

There were 82,087, 74,009, 75,472, 80,994, 75,963, and 74,591 OTUs in the UT13, UT27, UT33, UU13, UU27, and UU33 samples, respectively. The corresponding number of OTUs in the MT13, MT27, MT33, MU13, MU27, and MU33 samples were 69,376, 76,307, 79,951, 69,497, 77,290, and 79,130, respectively, while those in the LT13, LT27, LT33, LU13, LU27, and LU33 samples were 57,342, 55,941, 54,013, 58,308, 56,352, and 57,352, respectively.

**Figure 1:**
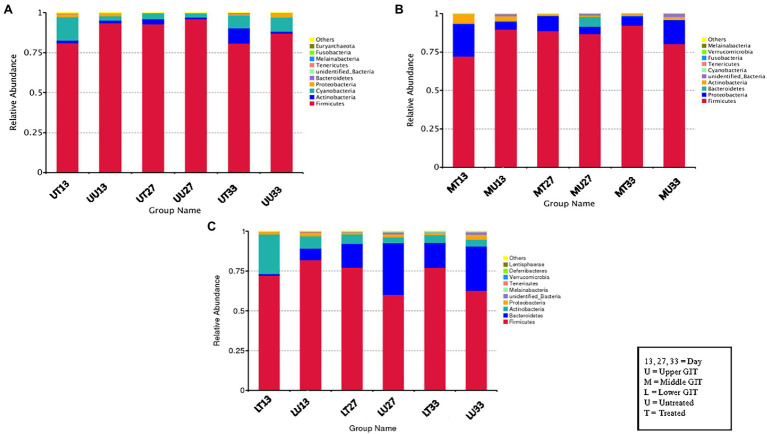
The top 10 phyla for each GIT section is shown, demonstrating the microbiome changes occurring in antimicrobial treated (T) and untreated (U) birds in the **(A)** upper (U), **(B)** middle (M) and **(C)** lower (L) GIT and following changes in feed type on day (D) 13, 27, and 33.

The top 10 phyla for each section of the GIT at the different sampling times for the untreated and treated birds is shown in Figure 1. The most common phylum was Firmicutes, which made up 60 to 96% of the bacterial composition, regardless of the sample type. Thereafter the most common phyla were Proteobacteria, Bacteroidetes, and Actinobacteria, which made up 1%–38%. However, the exact order varied depending on the GIT section, treatment/no treatment and/or the sampling time. Other phyla detected included Cyanobacteria, Tenericutes, Melainabacteria, Fusobacteria, Euryarchaeota, Verrucomicrobia, Deferribacteres, and Lentisphaerae.

**Figure 2:**
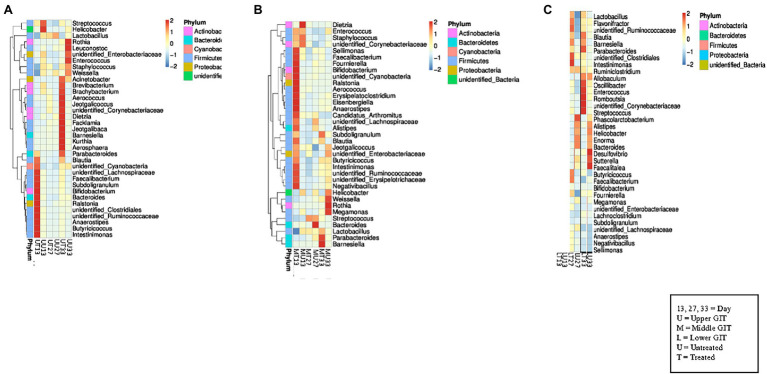
Heat maps showing the 35 top genera in each section of the GIT. **(A)** Shows the relationship between the top 35 genera in the upper GIT in both flocks at all sampling points, while **(B)** shows the middle GIT, and **(C)** shows the lower GIT.

In the upper GIT at the first time point *Blautia,* unidentified *Cyanobacteria,* unidentified *Lachnospiraceae, Faecalibacterium, Subdoligranulum, Bifidobacterium, Bacteroides, Ralstonia,* unidentified *Clostridiales,* unidentified *Ruminococcaceae, Anaerostipes, Butyricicoccus,* and *Intestinimonas* were detected at a higher concentration in the antibiotic treated birds (UT13) than in the samples from untreated (UU13), which contained *Streptococcus,* and *Helicobacter* in higher concentrations (Figure 2A). At the second sampling time similar genera were present in both the UT27 and UU27 samples, with no genera found to have a higher relative abundance between samples. At the final sampling *Acinetobacter, Brevibacterium, Brachybacterium, Aerococcus, Jeotgalicoccus,* unidentified *Corynebacterium, Dietzia, Facklamia, Jeotgalibaca, Barnesiella, Kurthia,* and *Parabacteroides* were detected at a higher concentration in the UT33 samples, while a greater amount of *Rothia, Leuconostoc*, unidentified *Enterobacteriaceae*, *Enterococcus, Staphylococcus,* and *Weissella* were found in the UU33 GIT contents. A similar pattern was observed in the middle GIT sections with *Sellimonas, Faecalibacterium, Bifidobacterium,* unidentified *Cyanobacterium, Ralstonia*, *Aerococcus, Erysipelatoclostridium, Eisenbergiella, Anaerostipes, Candidatus Arthomitus, Intestinimonas,* unidentified *Ruminococcaceae,* unidentified *Erysipelotrichaceae,* and *Negativbacillus* genera present at a higher concentration in the MT13 samples, while a higher concentration of the genus *Dietzia* was identified in the MU13 treatment group (Figure 2B). By day 27, with the exception of *Bacteroides*, which was elevated in treated samples, the same genera were detected in the treated and untreated middle GIT samples.

At day 33, the genera found in the treated and untreated samples were different, with *Parabacteroides* and *Barnesiella* present in a higher abundance in the MT33 samples and *Weissella, Rothia,* and *Megamonas* found in a larger quantity in the MU33 samples. Interestingly, there were fewer differentiating genera between treated and untreated samples in the lower GIT compared to the upper and middle sections. The only difference in the LT13 and LU13 samples was the presence of *Bifidobacterium* in the former and *Megamonas* in the latter (Figure 2C). The LT27 samples contained *Intestinimonas,* unidentified *Clostridiales,* and unidentified *Ruminococcaceae*, which was not found in the LU27 samples but *Phascolarctobacterium* was exclusively present in the latter. At final sampling, the LT33 samples had six genera (*Parabacteroides, Oscillibacter, Enterococcus, Romboutsia,* unidentified *Corynebacteriaceae,* and *Streptococcus*) that differentiated them from the LU33 samples. Moreover, these contained *Desulfovibrio, Satterella,* and *Faealitalea* that were not detected in the treated birds.

Despite these observed differences, analysis of the relative abundance of the top five phyla between treated and untreated birds in each GIT section at each time point indicated few significant differences were observed, except in the lower GIT where, at days 13 and 27 the relative abundance of Bacteroidetes was significantly decreased in treated birds in comparison with untreated birds (*p* < 0.005; Supplementary Figure S1). On the other hand the relative abundance of Actinobacteria was significantly increased in treated birds in the lower GIT on day 13 (*p* < 0.05). In the upper GIT there was a significantly greater relative abundance of Bacteroidetes on day 13 in treated birds (*p* < 0.0005).

A similar outcome was observed in the comparison of the relative abundance of the top five genera between treated and untreated birds in each GIT section at each time point, with the main changes being observed in the lower GIT. The relative abundance of *Bifidobacterium* was significantly greater (*p* < 0.05; Supplementary Figure S2) in treated birds on Day 13, while it was significantly greater (*p* < 0.05) for *Lactobacillus* on day 27. The relative abundance of the *Bacteroides* genus was significantly reduced (*p* < 0.005) in treated birds in the lower GIT at all three time points.

### Alpha Diversity Analysis

**Figure 3:**
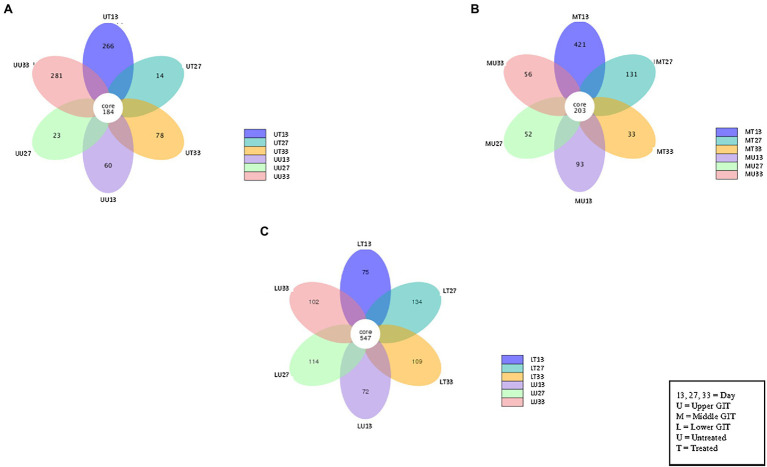
Flower Venn diagram depicting the shared and unshared OTUs between for the upper **(A)**, middle **(B)** and lower **(C)** GIT samples at each time point. Each petal represents a single sample group at a single time point. The number inside each petal represents the number of unique OTUs for that sample.

The number of shared and unshared OTUs are shown in Figure 3 for the upper (A), middle (B) and lower (C) GIT samples. Comparing treated vs. untreated broilers, the number of unshared OTUs in the upper GIT were 266 and 60, 14 and 23, and 78 and 281 at times 13, 27, and 33 days, respectively. The corresponding values for the middle GIT were, 421 and 93, 131 and 52, and 33 and 56, respectively, while in the lower GIT samples, unshared OTUs numbered 75 and 72, 134 and 114, and 109 and 102, respectively. Three indices were used to measure the alpha diversity in both flocks, in each section of the GIT, at every time-point. Wilcox and Tukey tests were performed on each diversity index in order to establish statistical significance (Table 2). Statistical significances (*p* ≤ 0.05) were observed in at least one diversity index in all of the following comparisons: the untreated flock vs. the treated flock at the same time-point (day 13, day 27, and day 33), the untreated flock at day 13 vs. day 27, and day 27 vs. day 33, and the treated flock at day 13 vs. day 27, and day 27 vs. day 33.

**Table 2:** Statistical significance of alpha diversity indices in each section of the GIT.

Samples/value of *p*	Upper GIT						Middle GIT						Lower GIT					
	ACE		Chao1		Shannon		ACE		Chao1		Shannon		ACE		Chao1		Shannon	
	T	W	T	W	T	W	T	W	T	W	T	W	T	W	T	W	T	W
U13-T13	0.08	0.00^*^	0.02^*^	0.00^*^	0.94	0.32	0.16	0.03^*^	0.10	0.03^*^	0.95	0.32	0.37	0.02^*^	0.29	0.03^*^	0.18	0.13
T27-T13	0.00^*^	0.00^*^	0.00^*^	0.00^*^	1.00	0.89	0.29	0.04^*^	0.21	0.04^*^	0.09	0.02^*^	0.00^*^	0.00^*^	0.00^*^	0.00^*^	0.00^*^	0.00^1^
T33-T13	0.01^*^	0.00^*^	0.00^*^	0.00^*^	0.33	0.05^*^	0.67	0.69	0.74	0.82	0.94	0.37	0.00^*^	0.00^*^	0.00^*^	0.00^*^	0.00^*^	0.00^*^
U27-U13	0.05^*^	0.00^*^	0.02^*^	0.00^*^	1.00	0.74	0.99	0.19	0.97	0.18	1.00	0.92	0.00^*^	0.00^*^	0.00^*^	0.00^*^	0.70	0.11
U33-U13	0.95	0.00^*^	0.82	0.00^*^	0.92	0.32	0.41	0.00^*^	0.20	0.00^*^	1.00	0.47	0.00^*^	0.00^*^	0.00^*^	0.00^*^	0.70	0.08
U27-T27	1.00	0.50	1.00	0.57	1.00	0.60	1.00	0.25	1.00	0.27	0.43	0.16	0.02^*^	0.01^*^	0.03^*^	0.01^*^	0.92	0.28
T33-T27	0.30	0.00^*^	0.17	0.00^*^	0.63	0.07	0.99	0.10	0.94	0.07	0.49	0.16	0.00^*^	0.00^*^	0.00^*^	0.00^*^	0.78	0.13
U33-U27	0.31	0.05^*^	0.29	0.06	0.79	0.19	0.80	0.02^*^	0.62	0.02^*^	1.00	0.54	0.00^*^	0.00^*^	0.00^*^	0.00^*^	1.00	0.89
U33-T33	1.00	0.05^*^	1.00	0.04^*^	1.00	1.00	0.93	0.05^*^	0.90	0.08	0.99	0.54	0.90	0.43	0.90	0.29	0.20	0.02^1^

T indicates the treated flock and U indicates the untreated flock. T, Tukey; W, Wilcox.

* Indicates *p* ≤ 0.05 (statistical significance).

### Beta Diversity Analysis

**Figure 4:**
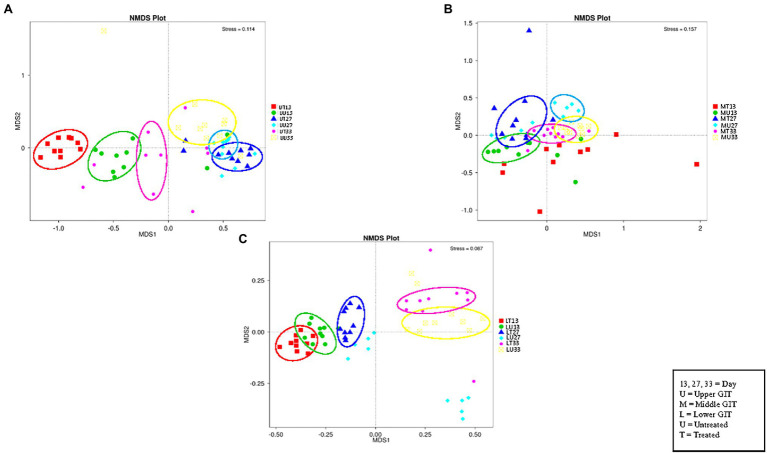
NMDS plots of the three GIT sections showing distinct clusters of OTUs in the upper **(A)**, middle **(B)**, and lower **(C)** GIT samples.

The NMDS plot (Figure 4) for the upper, (A), middle (B), and lower (C) GIT show six distinct clusters of OTUs in the upper and five in the middle and lower GIT samples. Thus, the OTUs obtained are distinct for each of the treated/untreated and sampling time combinations with the exception of the MT13 and MU27 samples.

**Figure 5:**
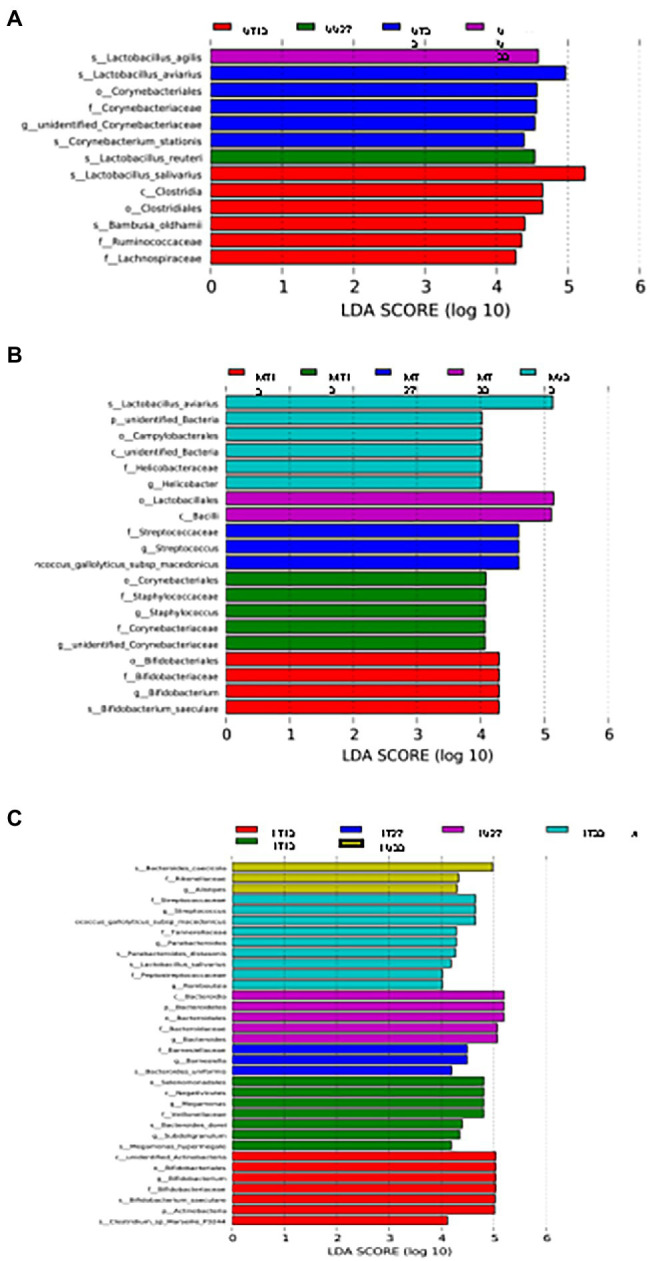
LEfSe analysis of the upper **(A)**, middle **(B)**, and lower **(C)** GIT.

LEfSe analysis was used to identify differential OTUs present between the samples (Figure 5). On day 13, the difference in the OTUs was attributed to the prevalence (presence and/or abundance) of *Lactobacillus salivarius*, *Clostridia*, *Clostridiales*, *Bambusa oldhamii*, *Ruminococcaceae*, and *Lachnospiraceae* in the UT13 samples, *Corynebacterium*, *Staphylococcus*, *Staphylococcaceae*, *Corynebacteriaceae*, and unidentified *Corynebacteriaceae* in MU13 samples, 6 different OTUs (3 *Bifidobacteriaceae*, unidentified *Actinobacteria*, Actinobacteria, and *Clostridium marseille*p-3244) in the LT13 samples, and 7 OTUs (*Selenomonadales*, *Negativicutes*, *Megamonas*, *Veillonellaceae*, *Bacteroides dorei*, *Subdoligranulim*, and *Megamonas hypermegale*.) in the LU13 samples. On day 27, the differentiating OTUs included *Lactobacillus agilis* in the UU27 samples, and 3 OTUs within the family *Streptococcaceae* in MT27 samples. On day 33 differentiating OTUs included *Corynebacterium stationis* in the UT33 samples, *L. agilis* in the UU33 samples, *Lactobacillus aviarius*, an unidentified bacterial phylum, *campylobacterales*, an unidentified bacterial class, and *Helicobacter* in MU33, and 2 OTUs (*Lactobacillales* and *Bacilli*) in MT33 (Figure 5B). On day 33, the differences in the lower GIT section were attributed to the prevalence of nine distinct OTUs (three *Streptococcaceae*, two *Parabacteroides, Tannerellaceae*, *L. salivarius*, *Peptostreptococcaceae*, and *Romboutsia*) in the LT33 samples and three OTUs in the LU33 samples (*Bacteroides cascicola*, *Rikenellaceae*, and *Allistipes*) (Figure 5C).

## Discussion

16S rRNA amplicon sequencing was used in this study to investigate changes in the broiler gastrointestinal tract (GIT) microbiome with age and antibiotic treatment. The limitations to this study included the sample size, the use of a single antibiotic, limited repetition and the absence of data on the microbiota of the broilers before treatment. Although 180 samples were analysed, this was divided into 18 different GIT section-treated/untreated-sampling time combinations and thus there were only 10 samples per combination. Sampling was also limited to three separate time points, when the birds were on starter, grower and finisher feeds. Thus the microbiota were influenced by the combination of antibiotic × age × feed type. While it could be argued that the control covers the age and feed factors, the two flocks were raised in separate broiler houses when ideally the treated and untreated birds would have been in the same broiler house as the specific environment will influence the microbiota (Kers et al., 2018). However, this was not possible in a commercial setting and the best that could be achieved was to have the two broiler houses on the same farm. This study examined the effect of one antibiotic, doxycycline, administered in drinking water at a single concentration of 100 mg/ml per kg body weight between days 8 and 12 thus the results only apply to this particular treatment strategy. Regardless of these limitations, the data generated, none-the-less, add to our growing knowledge and understanding of the broiler GIT microbiota including how this may vary depending on location, specific antibiotic treatment, age and/or feed.

The most common phylum was Firmicutes (regardless of GIT section or sampling time) while Cyanobacteria, Actinobacteria, Proteobacteria, and Bacteroidetes were also relatively abundant. Other studies have also reported that these phyla are abundant in the broiler GIT (Dragana et al., 2013; Feye et al., 2020). In contrast, the relative abundance of the different genera varied considerably depending on the section of the GIT and/or the sampling time, as previously reported (Dragana et al., 2014; Rychlik, 2020). These included *Lactobacillus,* known to be major colonisers of the upper GIT of broilers, where they digest starch and ferment lactate (Clavijo and Flórez, 2018) and *Ralstonia*, a phytopathogen associated with huge agricultural losses worldwide (Elsayed et al., 2020) and with nosocomial infections in immunocompromised patients (Fang et al., 2019).

The relative abundance of *Lactobacillus* (phylum Firmicutes, class Bacilli, order II Lactobacillales, and family Lactobacillaceae) and *Bifidobacterium* (phylum Actinobacteria, class Bacilli, order Bifidobacteriales and family Bifidobacteriaceae) varied over the course of this study, possibly due to the different composition of carbohydrates in the starter, grower and finisher feeds. The former are usually higher in corn based feeds while wheat favours *Bifidobacterium* (Shang et al., 2018).

As the birds got older, the relative abundance of *Lactobacillus* and *Weissella* (phylum Firmicutes, class Bacilli, order Lactobacillales and family Leuconostocaceae) increased in the upper and middle GIT. Di Cesare et al. (2019) made a similar observation and attributed this to the decrease in the crude protein concentration of the feed as the birds matured. However, changes in the fibre content in the feed may also play a role in bacterial selection, especially soluble fibres which act as prebiotics, promoting the growth of beneficial bacteria such as *Lactobacillus* and *Weissella* (Jha and Mishra, 2021).

When these bacteria digest finisher feed, they produce short chain fatty acids, which in turn, serve as an energy source for other bacteria. Thus, as previously observed by Walugembe et al. (2015), it was not unexpected that *Megamonas* (phylum Firmicutes, class Negativicutes, order Selenomonadales, and family Veillonellaceae), *Helicobacter* (phylum Proteobacteria, class Epsilonproteobacteria, order Campylobacterales, and family Helicobacteraceae) and *Rothia* (phylum Actinobacteria, class Actinobacteria, order Micrococcales, and family Micrococcaceae) were relatively abundant in the untreated flock on day 33.

Doxycycline is a broad-spectrum antibiotic in the tetracycline class of antibiotics and is used to treat colibacillosis, pasteurellosis, mycoplasmosis, and chlamydiosis in poultry (Cristina et al., 2010; Pavlova, 2015; Blau et al., 2019). *Bifidobacterium* (all samples), *Bacteroidetes* (upper GIT on day 13 and in the lower GIT on days 13, 27, and 33), *Enterobacteriaceae* (middle GIT on day 13) and *Lactobacillus* (upper and middle GIT) were more abundant in the treated vs. untreated birds. Environmental factors influence broiler microbiota (Kers et al., 2018) and may have contributed to the observed differences in the microbiota from the treated and untreated birds in our study. However, as the house design, broiler husbandry practices, biosecurity level, litter, feed access and climate were the same in the two broilers houses used, the impact of these factors was likely to be minimal.

Although comparative studies are scarce, Videnska et al. (2013) reported a decreased abundance of *Bifidobacteriales*, *Bacteroidales*, *Clostridiales*, *Desulfovibrionales*, *Burkholderiales*, and *Campylobacterales* and an increased prevalence of *Enterobacteriales* and *Lactobacillales* (specifically *Enterococcus* and *Escherichia*) in the faecal microbiota of broilers that received tetracycline therapy. Interestingly, this effect was reversed 24-h post treatment. (Maki et al., 2019) and the authors also observed increased shedding of *Enterococcus* and *Escherichia* in tetracycline treated broiler faeces.

In the present study, microbial diversity was significantly (*p* ≤ 0.05) affected by sampling time, antibiotic treatment and location within the GIT. Gao et al. (2017) suggested that antibiotic treatments delayed microbiota maturation while Dragana et al. (2014) previously reported significant differences in the microbiota of the different sections of the broiler GIT.

The NMDS and LEfSe analyses clearly demonstrated changes in the microbiota over time, possibly driven by changes in feed or maturation of the GIT. Our previous research suggested that the microbiota has a key role in establishing conditions that facilitate the survival and growth of *Campylobacter* (Greene et al., 2020). Although it may be coincidental, these pathogens are rarely detected in broilers before day 14 (Facciolà et al., 2017; Greene et al., 2020), which corresponds with the change in feed type from starter to grower and changes in the microbiota. *Campylobacter* is the most common cause of bacterial gastroenteritis globally with broilers the primary reservoir [26% of broilers and 38% of broiler carcasses within the European Union were found to be contaminated with *Campylobacter* in 2018 [EFSA (European Food Safety Authority), 2019]. Although not an objective of this research, our data suggests that further studies should be undertaken to investigate the link between age, diet, microbiota and *Campylobacter* carriage in broilers.

## Conclusion

It was concluded that the microbiota in the broiler GIT is influenced by doxycycline treatment but also the age of the birds and the location within the GIT. Future research should focus on gaining a better understanding of the factors such as gut maturation and changes in feed type that influence the microbiota and, in turn, how the microbiota can be manipulated to prevent carriage of human pathogens such as *Campylobacter*.

## Data Availability Statement

The datasets presented in this study can be found in online repositories. The names of the repository/repositories and accession number(s) can be found at: NCBI BioProject – PRJNA823705.

## Ethics Statement

The animal study was reviewed and approved by the Teagasc Animal Ethics Committee. Written informed consent was obtained from the owners for the participation of their birds in this study.

## Author Contributions

DB obtained the funding and was responsible for conceiving and the design of the study. GG acquired, analysed, and interpreted the data. GG, CB, and DB drafted the manuscript, while LK, PW, HL, AC, BL, and LO’C reviewed the manuscript. All authors contributed to the article and approved the submitted version.

## Funding

This work was funded by the Department of Agriculture, Food and Marine (DAFM), Ireland through the Food Institutional Research Measure (FIRM; Grant number 15F641). GG is a Teagasc Walsh Scholar (project number 2017262).

## Conflict of Interest

The authors declare that the research was conducted in the absence of any commercial or financial relationships that could be construed as a potential conflict of interest.

## Publisher’s Note

All claims expressed in this article are solely those of the authors and do not necessarily represent those of their affiliated organizations, or those of the publisher, the editors and the reviewers. Any product that may be evaluated in this article, or claim that may be made by its manufacturer, is not guaranteed or endorsed by the publisher.
