# Supplementary figures and images for: Effect of Doxycycline Use in the Early Broiler Production Cycle on the Microbiome
Source: Front Microbiol. 2022 Jul 7;13:885862. doi: 10.3389/fmicb.2022.885862 (PMC9301238; doi:10.3389/fmicb.2022.885862)

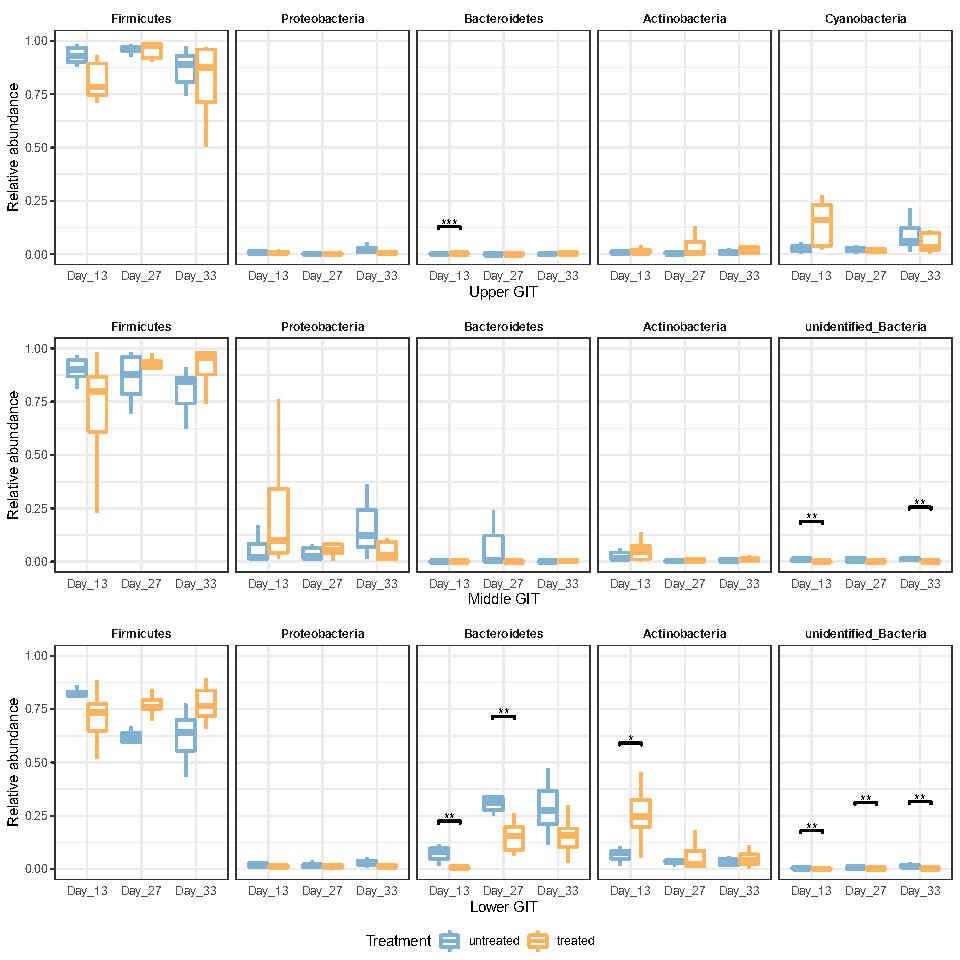

Supplement: Supplementary file 1 [file Image_1.JPEG]

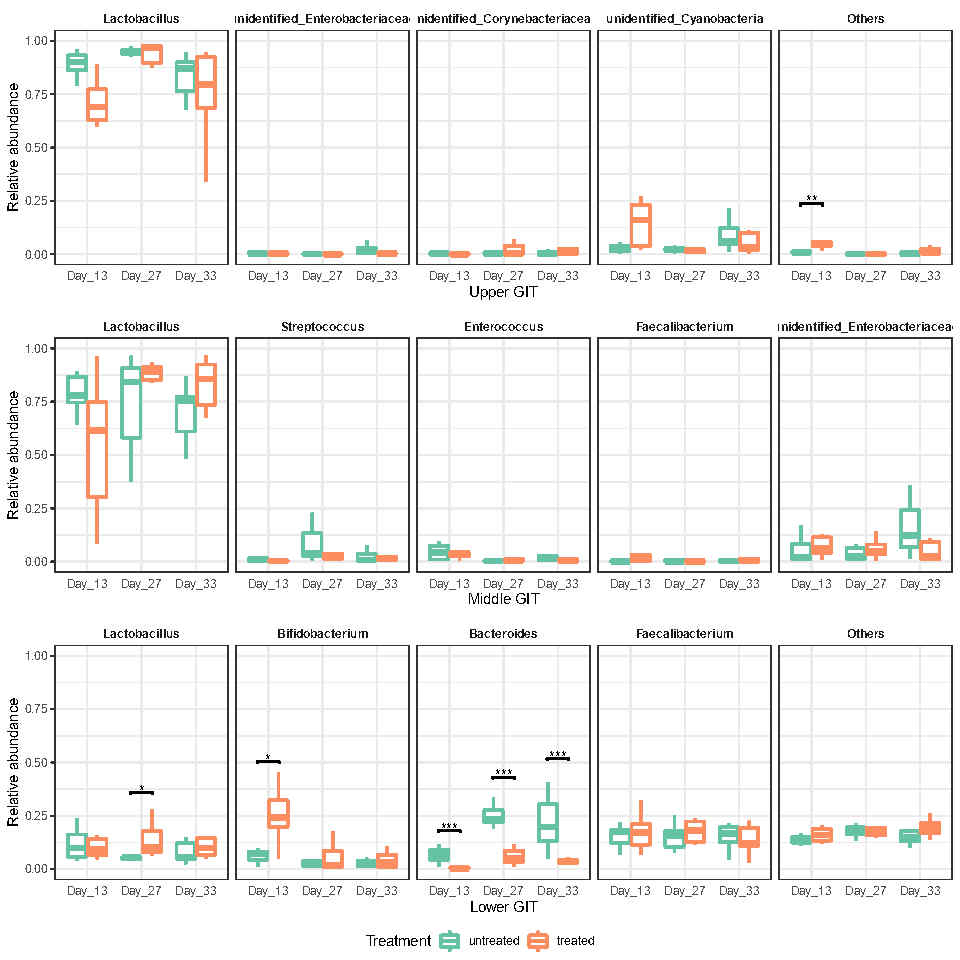

Supplement: Supplementary file 2 [file Image_2.JPEG]
